# Risk of myocardial infarction and overall mortality in survivors of venous thromboembolism

**DOI:** 10.1186/1477-9560-6-10

**Published:** 2008-08-18

**Authors:** Consuelo Huerta, Saga Johansson, Mari-Ann Wallander, Luis A García Rodríguez

**Affiliations:** 1 Spanish Centre for Pharmacoepidemiologic Research (CEIFE), Madrid, Spain; 2 AstraZeneca R&D Mölndal, Sweden; 3 Institute of Medicine, Sahlgrenska Academy, Gothenburg University, Sweden; 4 Department of Public Health and Caring Science, Uppsala University, Sweden

## Abstract

**Background:**

Venous thromboembolism (VTE) and thromboembolic arterial diseases are usually considered to be distinct entities, but there is evidence to suggest that these disorders may be linked. The aim of this study was to determine whether a diagnosis of VTE increases the long-term risk of myocardial infarction (MI).

**Methods:**

The incidence rate (IR) and relative risk (RR) of MI in a cohort of patients with a diagnosis of VTE (n = 4890) compared with that of a control cohort without prior VTE (n = 43 382) were evaluated in the UK General Practice Research Database (GPRD). Death during follow-up was also determined. Patients were followed for up to 8 years (mean of 3 years).

**Results:**

The IR of MI per 1000 person-years was 4.1 (95% CI: 3.1–5.3) for the VTE cohort and 3.5 (95% CI: 3.2–3.8) for the control cohort. The IR of MI was highest in the first year after the VTE episode, but overall differences between the two cohorts were not significant (RR of MI associated with VTE: 1.2; 95% CI: 0.9–1.6). The risk of death was higher in the VTE cohort than the control cohort, even after adjustment for cancer, heart failure and ischaemic heart disease (RR: 2.4; 95% CI: 2.2–2.6), particularly during the first year after VTE (RR: 3.8; 95% CI: 3.4–4.3).

**Conclusion:**

A VTE episode does not significantly increase the risk of MI, but does increase the risk of death, particularly in the first year following VTE diagnosis.

## Background

Venous thromboembolism (VTE), usually manifested as deep vein thrombosis (DVT) or pulmonary embolism (PE), is usually considered to be a distinct entity from the thromboembolic arterial diseases, such as myocardial infarction (MI), peripheral artery disease and ischaemic stroke. However, both VTE and thromboembolic arterial diseases involve the formation of clots within blood vessels, and so may be linked. Recent studies have investigated the incidence of arterial events in patients with VTE, and have reported data suggesting that there may be a positive association between VTE and thromboembolic arterial disease [1-3]. However, these studies either lacked a suitable control group or involved relatively small numbers of patients.

To determine whether a diagnosis of VTE is associated with an increased risk of arterial disease, the incidence of arterial disease in patients with a prior VTE should be compared with that in matched controls without a history of VTE. In this study we aimed to perform such an analysis by assessing the risk of MI in patients with a VTE diagnosis and control patients without VTE using data recorded prospectively from the UK General Practice Research Database. We also aimed to estimate the overall long-term mortality among patients with VTE during a long-term follow-up period in comparison with that of the control population without VTE.

## Methods

### Data source

The GPRD contains computerized information entered by primary care physicians (PCPs) in the UK. The vast majority of the UK population is registered with a PCP. About 1500 PCPs participate in the GPRD, covering a population of around 3 million individuals, who are broadly representative of the UK population. The PCPs hold the complete medical record of registered individuals, including demographic data, all medical diagnoses, consultant and hospital referrals, and a record of all prescriptions issued. Prescriptions are generated directly from the PCP's computer and entered into the patient's computerized file. All the information is recorded by PCPs during consultations in a standard fashion and practices regularly anonymize and send these data to the Medicines and Healthcare Products Regulatory Agency (MHRA), which is in charge of quality control and management of the data for use in research projects. Several validation studies have shown the accuracy and completeness of data in the GPRD [4,5]. Previous studies have also confirmed the validity of using the GPRD for epidemiological research in the field of DVT and PE [6-10].

### Study cohorts

The source population included individuals aged 20–79 years enrolled with a participating PCP for more than 2 years during 1 January 1994 to 31 December 2000, without a previously recorded diagnosis of VTE, as described in a previous cohort study of the natural history of VTE [11]. The resulting source population consisted of 1 856 206 patients, and the first day of meeting these eligibility criteria was used as each individual's start date. Of this source population, 6550 patients had a first recorded diagnosis of VTE from the individual's start date until 31 December 2000. The validation (positive predictive value) of a VTE diagnosis in the GPRD has been described previously: a questionnaire was sent to PCPs for a random sample of 5% of patients with a record of VTE and, after reviewing these questionnaires, the diagnosis of VTE was confirmed in 94% of cases [11]. Moreover, we previously reported that the overall incidence rate of VTE in the study cohort was 74.5 per 100 000 person-years [11]. In other epidemiological studies, the incidence of VTE ranged from 71 to 117 case per 100 000 of the population per year (standardized for age and sex) [12].

We classified VTE episodes as idiopathic if they occurred in the absence of the following transient risk factors: fracture, surgery, pregnancy or childbirth, or any hospitalization (all occurring in the 3-month period before VTE); cancer in the year before VTE; or use of hormone replacement therapy or oral contraceptives in the 6 months before the date of the VTE episode. We considered all other cases of VTE to be secondary, in a similar manner to other studies [1,13].

A control cohort was also identified. For this, 50 000 individuals without a VTE diagnosis were randomly sampled from the source population and matched by age, sex and calendar year to the VTE cohort.

### Definition of clinical endpoints

#### Myocardial infarction

Patients with a history of ischaemic heart disease prior to their start date were excluded from both VTE and control cohorts for the analysis of the risk of MI following a VTE, as a history of ischaemic heart disease could mask the influence of VTE on a subsequent MI. Each individual's start date was the date of diagnosis of VTE in the VTE cohort and a random date within the study period for the control cohort. Follow up started a month after the start date in order to exclude patients dying due to the initial episode of VTE. Finally, there were 4890 patients in the VTE cohort and 43 382 patients in the control cohort.

Patients in both cohorts were then followed up until one of the following endpoints was reached: a recorded code of MI, age of 80 years, death or the end of the study period (31 December 2002). We manually reviewed the computerized profiles of all patients identified with a code of MI, and all deaths. We considered MI cases to be patients whose diagnosis was confirmed by a letter from a consultant cardiologist or on hospital discharge. We also considered as cases: those who died from coronary heart disease (CHD); patients with post-mortem evidence of a recent MI or a recent coronary artery occlusion; patients with ante-mortem evidence of CHD in the absence of another cause of death; and patients for whom CHD was recorded as the underlying cause of death. We did not contact PCPs for further confirmation of the diagnosis of MI, as our experience from a previous study of MI in the GPRD has shown that case ascertainment after manual review of the computerized information supports our case definition in more than 90% of instances [14].

#### Mortality

For the mortality analysis, individuals with a history of ischaemic heart disease prior to the start date were not excluded, but follow-up was started 1 month after the episode of VTE as before. (Data for patients who died within the first month of the VTE diagnosis have been presented elsewhere [11].) The VTE cohort consisted of 5801 patients and the control cohort consisted of 48 399 patients. Patients were followed until death, age of 80 years or the end of the study period (31 December 2002).

### Analysis of MI risk

#### Relative risk and Kaplan-Meier survival analysis

Estimates of MI occurrence (with 95% CI) were calculated for the VTE and control cohorts. Individuals alive at the end of the study period were regarded as censored from that date, while individuals with their last practice visit before the end of the study were regarded as censored from the date of their last practice visit. The cumulative hazard of MI was calculated using a Kaplan-Meier survival analysis. Cox proportional hazards regression was used to estimate the relative risk (RR) and 95% confidence intervals of MI in the VTE cohort compared with the control cohort (overall and according to type of VTE). Variables included in the multivariate model were the presence of heart failure and hypertension, as well as frequency-matched variables (age, sex, and calendar year).

### Analysis of mortality

Deaths from any cause during the follow-up period were analysed using Kaplan-Meier life-tables to compare survival between patients with or without VTE. Cox proportional hazards regression was used to estimate the RR and 95% CI of death in the VTE cohort compared with the comparison cohort. Variables included in the multivariate model were the presence of cancer or heart failure as well as the frequency-matched variables (age, sex and calendar year). All statistical analyses were conducted using STATA (version 8.2; Stata Corporation, College Station, Texas, USA).

## Results

### Risk of myocardial infarction

The incidence and risk of MI were determined for the cohort of VTE patients (n = 4890) and the control cohort (n = 43 382), in order to provide an estimate of the RR of MI following VTE. The age distribution in the two cohorts was successfully matched, with 12.3% of both cohorts aged 20–39 years, 33.3% aged 40–59 years, 27.1% aged 60–69 years and 27.3% aged 70 years or older. During the mean follow-up period of 3 years (range: 3–8 years; median: 3 years), MI occurred in 55 patients from the VTE cohort and 472 patients from the control cohort. Thus the incidence rate (IR) of MI per 1000 person-years was 4.1 (95% CI: 3.1–5.3) for the VTE cohort and 3.5 (95% CI: 3.2–3.8) for the control cohort. The difference between the two groups was not significant, as shown by the RR of MI (RR: 1.2; 95% CI: 0.9–1.6). The incidence rates of MI were within the range reported in previous population-based studies in the UK (2.73–8.23 and 0.66–2.56 per 1000 person-years in men and women, respectively [15,16]).

**Figure 1:**
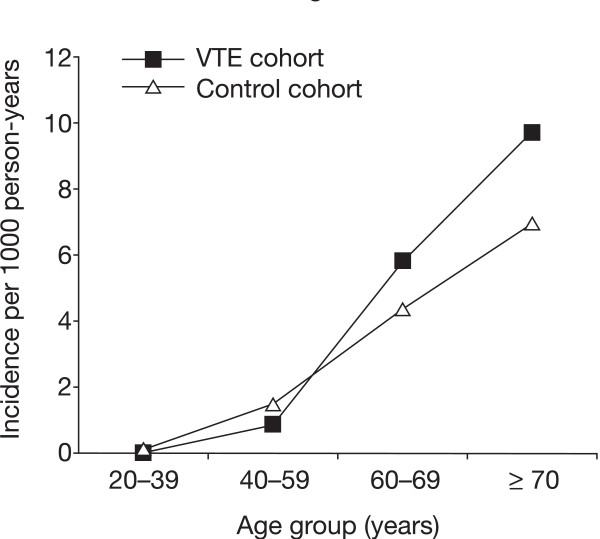
**Incidence rate (IR) and relative risk (RR) of myocardial infarction in the venous thromboembolism (VTE) cohort and control cohort according to age**.

**Figure 2:**
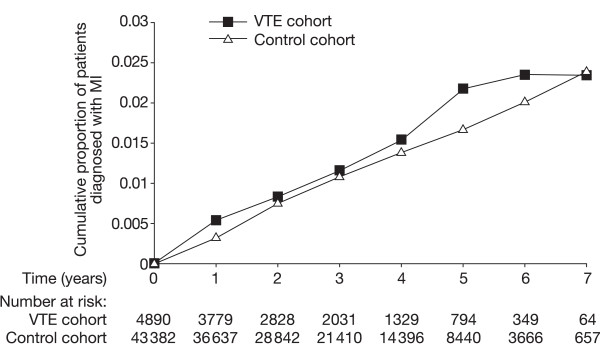
Cumulative proportion of patients diagnosed with myocardial infarction (MI) in the venous thromboembolism (VTE) cohort and control cohort over time (log-rank test > 0.05).

The IR of MI increased with age in both cohorts (see Figure 1). Although the IR of MI was numerically greater in the VTE cohort than the control cohort for those aged 60–69 years or at least 70 years, determination of RR indicated that these differences were not significant (RR: 1.3; 95% CI: 0.8–2.0 for patients aged 60–69 years, RR: 1.4; 95% CI: 0.9–2.0 for those aged 70 years or more). The cumulative proportion of patients diagnosed with MI over time for the two cohorts is shown in Figure 2. This analysis showed that the cumulative proportion of patients with MI was slightly greater in the VTE cohort than the control cohort in the first year, but this difference narrowed in years 2–4. Indeed, the increased risk of MI in the VTE cohort compared with the control cohort in the first year was of borderline significance (adjusted RR: 1.6; 95% CI: 1.0–2.5). After the first year, the adjusted RR of MI associated with VTE fell to 1.0 (95% CI: 0.7–1.5).

Further analysis of the risk of MI in the VTE cohort compared with the control cohort for the first year showed that the excess risk was of borderline significance in patients aged between 60 and 69 years (RR: 2.0; 95% CI: 1.0–4.0) and insignificant in the younger age group (40–59 years of age, RR: 0.70; 95% CI: 0.1–5.3) and older age group (≥70 years, RR: 1.5; 95% CI: 0.8–2.9). The risk of MI in the VTE cohort, however, was similar for patients who had had DVT (n = 33) and for patients who had had PE with or without DVT (n = 22) (Table 1). The risk of MI was also similar regardless of whether the VTE was idiopathic or secondary (Table 1).

**Table 1:** Incidence rate and relative risk of myocardial infarction in the venous thromboembolism cohort compared with the control cohort in the first year of follow up.

	**Myocardial** **infarction** ** cases (n = 159)**	**Incidence** **rate per 1000** ** person-years**	**Relative risk** ** (95% CI)^a^**
**Control cohort (n = 43 382)**	136	3.4 (2.9–4.0)	1
**Venous thromboembolism cohort (n = 4890)**	23		
Deep vein thrombosis	15	5.7 (3.4–9.4)	1.7 (1.0–2.9)
Pulmonary embolism	8	4.8 (2.4–9.6)	1.5 (0.7–3.1)
Secondary venous thromboembolism	13	5.2 (3.0–8.9)	1.6 (1.0–2.8)
Idiopathic venous thromboembolism	10	5.6 (3.0–10.4)	1.6 (0.8–3.2)

^a^Adjusted relative risk derived from Cox regression models including sex, age, calendar year, heart failure, hypertension, and smoking.

### Mortality

**Figure 3:**
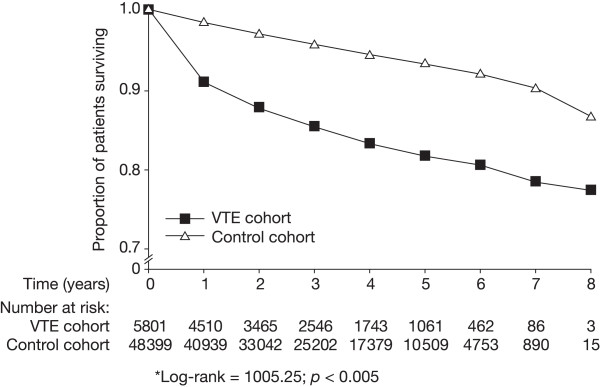
Kaplan-Meier survival curves for venous thromboembolism (VTE) cohort and control cohort (*log-rank = 1005.25; p < 0.005).

During the total follow-up period of 8 years, 3088 patients died: 2266 of 48 399 in the control cohort and 822 of 5801 in the VTE cohort. Overall mortality was, therefore, higher in the VTE cohort (14.2%; 49.5 per 1000 person-years) than the control cohort (4.7%; 14.5 per 1000 person-years) (Figure 3). After adjustment for the presence of cancer, ischaemic heart disease and heart failure, the RR of death during this 8-year period in the VTE cohort compared with the control cohort was 2.4 (95% CI: 2.2–2.6). When we re-analyzed the mortality findings after excluding patients with a history of ischaemic heart disease to allow comparison with the incidence of MI in these cohorts, the mortality rates dropped to 47.4 and 12.1 per 1000 person-years in the VTE and control cohorts, respectively.

The increased risk of death associated with VTE was much greater in the first year after VTE diagnosis (RR: 3.8; 95% CI: 3.4–4.3) than in subsequent years (RR: 1.6; 95% CI: 1.8–1.4) (Table 2). The risk of death in the first year was also greater in patients with a diagnosis of DVT (RR: 4.4; 95% CI: 3.9–5.1) than in those with a diagnosis of PE (RR: 2.9; 95% CI: 2.5–3.5) (Table 2). Compared with the control group, mortality was increased in patients aged 20–59 years (RR: 10.5; 95% CI: 7.3–15.1) and at least 60 years (RR: 3.1; 95% CI: 2.7–3.6).

**Table 2:** Mortality and relative risk of MI in the venous thromboembolism cohort compared with the control cohort, according to year of follow up.

	**First year**			**After 1 year**		
	**Deaths** ** (n = 1216)**	**Mortality/1000** **person-years** ** (95% CI)**	**RR (95%** ** CI)**^a^	**Deaths** ** (n = 1872)**	**Mortality/1000** **person-years** ** (95% CI)**	**RR (95% CI)**^a^

**Control cohort**	716	16.0 (14.9–17.1)	1	1550	13.9 (13.2–14.6)	1
**All VTE cases**	500	97.8 (89.6–106.8)	3.8 (3.4–4.3)	322	28.1 (25.2–31.3)	1.6 (1.4–1.8)
DVT	344	113.4 (102.0–126.1)	4.4 (3.9–5.1)	186	27.7 (24.0–32.0)	1.6 (1.4–1.9)
PE	156	75.1 (64.2–87.9)	2.9 (2.5–3.5)	136	28.7 (24.2–33.9)	1.6 (1.3–1.9)

CI, confidence interval; DVT, deep vein thrombosis; PE, pulmonary embolism; RR, relative risk; VTE, venous thromboembolism.

^a^Relative risk estimated by Cox regression adjusted for age, sex, calendar year, consultations in the previous year, cancer, heart failure and ischaemic heart disease.

Causes of death for patients dying within the first year of follow up are shown in Table 3. The main cause of death was cancer in both groups, but the percentage of patients dying from cancer was almost two-fold higher in the VTE group (56.0 vs. 29.6%). Conversely, the proportion of patients dying from CHD was approximately two-fold greater in the control cohort than the VTE cohort.

**Table 3:** Distribution of causes of death in patients dying during the 11 months of follow up in patients with VTE surviving the first month after VTE diagnosis in comparison with control cohort.

**Cause of death**	**VTE cohort (n = 500)** ** n (%)**	**Control cohort (n = 716)** ** n (%)**
**Coronary heart disease (CHD)**	49 (9.8)	153 (21.4)
**Other cardiovascular and cerebrovascular diseases**	63 (12.6)	107 (14.9)
**Cancer**	280 (56.0)	212 (29.6)
**Other non-cardiovascular diseases (respiratory, digestive, urinary, other)**	47 (9.4)	129 (18.0)
**Unknown**	61 (12.2)	115 (16.1)

## Discussion

The results of this study suggest that a first VTE episode does not increase the risk of MI. These results were similar, regardless of VTE type (DVT or PE), or whether VTE was idiopathic or secondary. However, while we did not observe a significant increase in the risk of MI following VTE (RR: 1.2 with a lower 95% CI below 1.0), the upper 95% confidence interval of 1.6 means that an increased risk of MI following VTE cannot be safely excluded on the basis of our results.

These results contrast with several, much smaller, studies that have pointed towards an association between VTE and thromboembolic arterial disease. Case-control studies have reported a significantly higher prevalence of carotid plaques in patients with DVT (n = 299) [17] and a significantly higher incidence of coronary artery calcification in patients with idiopathic VTE (n = 89) compared with matched controls lacking VTE [18]. Moreover, Bova and colleagues found a significantly higher risk of arterial events in 151 patients with VTE compared with 151 controls (HR 2.9; 95% CI: 1.1–7.6) [13]. Recently, a cohort study reported the risk of MI to be increased by 60% in the first year after an episode of VTE, with a progressive decline during the subsequent 20 years [19]. As the present study includes nearly 5000 patients with VTE and a control cohort without prior VTE (n = 43 382), our conclusion that VTE is not associated with a major increased risk of subsequent MI is likely to be authoritative.

The conclusions of our study contradict those of Bova and colleagues [13], but this may reflect, in part, differences in the criteria used to identify patients with VTE and the arterial events used as endpoints. Moreover the latter study was comparatively small, though to our knowledge it is the only study other than ours and the aforementioned study by Hong et al. in 89 patients with VTE [18] to compare patients with VTE with controls taken from a general population without VTE. Other studies investigating VTE as a risk factor for cardiovascular events did not include a control group without VTE. For example, two studies compared patients with idiopathic VTE with patients diagnosed with secondary VTE [1,2], and one investigated the long-term effects of 6 weeks vs 6 months of anticoagulation treatment on patients with VTE [3]. Given the large number of patients with first VTE (n = 4890) in our study, it seems unlikely that VTE is associated with a subsequent MI for patients without a history of ischaemic heart disease.

In contrast to the conflicting results surrounding the association between DVT and cardiovascular disease, it is well recognised that there is an increased mortality after VTE [3]. The present study showed that a first diagnosis of VTE was associated with significantly increased mortality in those who survived the first month following the VTE event, particularly in the subsequent 11 months after VTE diagnosis. This risk was greatest in patients with DVT rather than PE, and in younger patients rather than the elderly. Previous studies have shown that mortality is highest immediately after VTE in patients with PE, and then decreases over the following year [20,21]. However, to our knowledge, few studies have investigated mortality in patients for prolonged periods after VTE. Our study therefore provides important data on long-term mortality following a VTE event in a large number of patients (n = 5801). These findings complement those from a previous study in the same cohort of patients with VTE which reported patient mortality in the first month after VTE [11].

The present study showed that, during the first year after VTE, cancer was the most frequent cause of death in VTE patients surviving the first month. Cancer is a well-known risk factor for VTE and death from VTE [3,11,22-24], with the mortality seemingly independent of whether the cancer diagnosis is made before or after VTE diagnosis [24]. As such, cancer (and death because of cancer) is likely to be more common in the VTE cohort. Secondly, the data for this period may be skewed somewhat, as the 1-month period after VTE is omitted. During this 1-month period there were more deaths due to cardiovascular causes than cancer, as has been reported previously [11]. Deaths during the 1-month period after VTE were largely due to PE, rather than DVT, with the 1-month death rate of 1.4% after an episode of DVT and 22.6% after PE with or without DVT [11].

The 8-year risk of death in patients surviving the first month after VTE is higher than that in patients without VTE, even after adjustment for cancer, heart failure and ischaemic heart disease. Because of the likely multiple comorbid diseases and risk factors in the predominantly elderly population with VTE in this study, we should be cautious about the reasons for this excess mortality. Thus, although the 8-year risk of death in patients surviving 1-month after VTE is higher than that in patients without VTE, it is difficult to say whether this increased risk arises from VTE itself or other underlying conditions or risk factors.

This study has a number of important strengths. Patients were drawn from a large primary care database representative of the UK population and spanning a wide age range, and representing a study population that is an order of magnitude larger than previous studies investigating potential risk factors and complications of VTE [13,17,18,25-29]. Cases of VTE were classified according to whether they were DVT or PE, and also if they were idiopathic or secondary, allowing analysis of possible differences between the types of VTE. VTE cases in a random sample were also identified and validated with a confirmation rate of over 94% [11]. We excluded information bias since information was collected in the same manner for VTE and comparison patients, and information collection in VTE cases was blinded to the later occurrence of MI. Using MI, a major clinical event, as the cardiovascular endpoint was advantageous as previous studies using the GPRD have shown the validity of using codes for MI [14,30]. Limitations of the study include the fact that it only involved a UK population sample and that patients included in the analysis of MI risk may have had subclinical cardiovascular disease prior to the start date of the study. Patients may also have had VTE or MI prior to enrolment in the GPRD, which would not have been systematically recorded. In addition, the limited number of MI cases in the VTE cohort (55) means that the study is not powered to detect modest but potentially clinically important elevations in the incidence rate of MI.

## Conclusion

In conclusion, our data show that a diagnosis of VTE does not increase the risk of MI in comparison with a control cohort drawn from the general population of the UK. There was some suggestion that the risk of MI may be increased in elderly patients with VTE during the first year following the diagnosis of VTE, but this increase was not statistically significant. While the risk of MI is not increased, patients who survive the first month after a VTE event were significantly more likely than controls to die in the subsequent 8 years. This increased risk is particularly marked over the first year following a VTE. Further studies may thus be required to investigate the causes of death following VTE in more detail, and thus determine how best to reduce mortality in patients after a first VTE event.

## Competing interests

This study was funded by a research grant from AstraZeneca R&D Mölndal, Sweden. SJ is an employee of AstraZeneca R&D Mölndal, Sweden, and MAW was an employee of AstraZeneca R&D Mölndal, Sweden at the time of the study. The corresponding author had full access to all the data in the study, and takes responsibility for the integrity of the data and the accuracy of the data analysis.

## Authors' contributions

CH participated in the design of the study, carried out the statistical analysis, interpreted the data and helped to draft the manuscript. SJ and MAW participated in the design of the study and interpretation of the data. LAGR conceived of the study, participated in its design, analysis and interpretation and helped to draft the manuscript. All authors critically revised the manuscript for important intellectual content, and approved the final manuscript.
